# Functional Involvement of a Mitogen Activated Protein Kinase Module, OsMKK3-OsMPK7-OsWRK30 in Mediating Resistance against *Xanthomonas oryzae* in Rice

**DOI:** 10.1038/srep37974

**Published:** 2016-11-29

**Authors:** Siddhi Kashinath Jalmi, Alok Krishna Sinha

**Affiliations:** 1 National Institute of Plant Genome Research, Aruna Asaf Ali Marg, New Delhi, 110067, India

## Abstract

Mitogen-activated protein kinases (MAPKs) are highly conserved signaling modules in eukaryotes, transmitting signals from upstream receptor to downstream target by phosphorelay mechanism. Here we report involvement of a poorly characterized group C MAPK of rice namely, OsMPK7 along with its upstream MAPK kinase, OsMKK3 and downstream target, OsWRKY30 during *Xanthomonas oryzae* infection, a causal agent of leaf blight disease in rice. *X. oryzae* infection resulted in induction of *OsMPK7* and *OsMKK3*. OsMKK3 was found to physically interact and phosphorylate OsMPK7. Overexpression of OsMPK7 and OsMKK3, individually and in combinations resulted in inhibition of disease symptoms caused by *X. oryzae*, however silencing of OsMPK7 resulted in disease susceptibility. Furthermore, OsWRKY30 was identified as downstream target of OsMPK7 through protein-protein interaction techniques and was found to be a positive regulator of defence response against *X. oryzae* pathogen. The overexpression of OsMKK3-OsMPK7 upregulated genes involved in pathogenesis, cell wall structure maintenance and cell metabolism indicating possible mechanism of disease resistance. These leaves also showed restricted movement of the pathogen from the point of infection to uninfected area. Taken together, this work suggests a positive involvement of OsMKK3-OsMPK7-OsWRKY30 module in imparting disease resistance against *X. oryzae* infection in rice.

Plants posses several integrated signaling networks which predominantly involve protein kinases that perceive and respond to the different stimuli. One of the most important protein kinase cascades that transfer extracellular signals into intracellular responses is the highly distinct and conserved mitogen activated protein kinase (MAPK) cascade. The MAPK cascade comprises a linear cascade of three consecutively acting protein kinases, namely MAPKK Kinases, MAPK Kinases and MAPKs, connected to each other by an event of phosphorylation. The sequential activation of the MAPK cascade ultimately results in activation of myriad of proteins leading to expression of specific sets of genes in response to environmental stimuli^1^,^2^. MAPKs are activated by dual phosphorylation of conserved threonine (T) and tyrosine (Y) residues in the motif TXY located in their activation loop by MAPKKs, which are themselves activated by MAPKKKs through phosphorylation of conserved serine (S) and/or threonine (T) residues in S/T-X_3-5_-S/T motif^1^,^3^. The completion of rice genome project revealed 15 MAPKs and 8 MAPKKs, whereas *in silico* analysis of rice genome database revealed 75 MAPKKKs^4^,^5^,^6^. The MAPKs are divided into 4 groups based on protein structure and sequence motif “TXY”. The MAPKs having sequence motif of “TEY” are grouped into A, B and C whereas those having “TDY” sequence are organised into D group. In rice, the most studied members, OsMPK3 and OsMPK6 belongs to Group A and OsMPK4 belongs to Group B^7^,^8^. However, the information is quite scant regarding the group C members comprising of OsMPK7 and OsMPK14. The other ten members fall in group D, the function of all these also remains elusive^5^.

Upon microbial attack plants are equipped to sense and mount a defence response against pathogens due to the presence of specific receptors and signaling cascades^1^,^9^,^10^. Detection of Pathogen Associated Microbial Patterns (PAMPs) by membrane Pattern Recognition Receptors (PRRs) triggers early defence responses like activation of MAPKs, calcium flux and production of reactive oxygen species (ROS). These early events then causes activation of intermediate and late defence responses like activation of defence genes, strengthening of cell wall, phytoalexin biosynthesis, hypersensitive response and induced resistance^9^,^10^,^11^. ROS burst associated with PTI is known to activate MAPKs which in turn regulate ROS production^12^,^13^. It has been examined that moderate concentration of ROS is essential in the regulation of biological processes, whereas its high concentrations result in oxidative stress and causes irreversible damage seen as typical sign of infection^14^.

Arabidopsis MAPKs are known for prominent role in pathogen signaling^10^. MEKK1-MKK4/5-MPK3/6 in Arabidopsis is activated in response to PAMPs like bacterial flg22 elicitor, EF-Tu, Chitin, PGNs, which ultimately leads to the transcriptional activation of several downstream targets like flg22-INDUCED RECEPTOR- LIKE KINASE 1 (FRK1), WRKY22, WRKY29, ACS2/6, NIA2, ERF104 and VIP1 leading to defence responses^10^,^15^. Another MAPK module MEKK1-MKK1/2-MPK4 is considered to negatively regulate defence responses where MPK4 acts on its downstream substrates, MAP Kinase substrate 1 (MKS1) and WRKY33^16^,^17^. Studies in Arabidopsis reports that MKK3 interact and activate MPK6 and MPK7 in response to Jasmonic acid and *Pseudomonas syringae*, respectively which further activates pathogenesis related genes^18^,^19^.

In comparison to Arabidopsis, rice MAPKs have been partially characterized. A couple of studies performed to find interaction between rice MAPKs and MAPKKs have put forward some of the major MAPK modules of which only a few are functionally characterized^8^,^20^. A cascade consisting of OsMKK4-OsMPK3/6 has shown its negative role in MAMP-triggered immunity and positive regulator of drought, salt and cold stress^20^. OsMKK6 has been reported to be involved in regulating genes for biosynthesis of phytoalexin, a secondary metabolite known to get accumulated in *Magnoporthe oryzae* infected plants^21^. Interestingly, a unique interaction was reported among two MAPKs in rice, OsMPK3 and OsMPK20-4 that showed resistance against *P. syringae* infection when transiently expressed in tobacco leaves^22^. Moreover, a recent study reports a unique role of rice MPK3 in imparting submergence tolerance regulated by SUB1A1 by a positive feedback loop^23^. However, involvement of rice MAPKs particularly of group C in pathogen signaling still remains elusive. To date there is only a single report of the involvement of group C MAPKs in circadian rhythm^24^.

*X. oryzae*, a gram negative bacteria, known to cause leaf blight is the most serious disease in rice affecting its yield^25^,^26^. In the current manuscript we have investigated the role of a least studied member of rice group C MAPK namely, OsMPK7 during *X. oryzae* infection. Rice leaves and roots overexpressing OsMPK7 showed resistance while OsMPK7 silencing showed susceptibility towards *X. oryzae* infection. This work provides OsMKK3-OsMPK7-OsWRKY30 module in regulating disease resistance against *X. oryzae*.

## Results

### *X. oryzae* infection in rice induces OsMPK7

**Figure 1:**
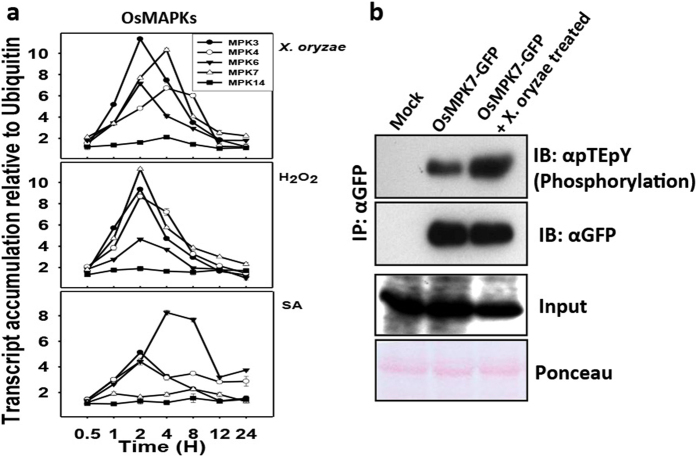
Quantitative evaluation of rice MAPKs transcript level and activation of OsMPK7 by *X. oryzae*. (**a**) Transcript levels of MAPK genes were examined in 15 days old rice seedlings in response to *X. oryzae*, H_2_O_2_ and Salicylic acid (SA) treatment by Q-RT-PCR. Ubiquitin and actin gene was considered as internal control. The experiments were repeated three times with three technical replicates. (**b**) OsMPK7 phosphorylation was determined through immunological assay by immunoprecipitation of OsMPK7-GFP fusion protein using anti-GFP antibody from 50 D old rice leaves overexpressing OsMPK7 fusion protein. Immunoblotting was carried out using pTEpY antibody to detect phosphorylated MAPK TEY domain upon *X. oryzae* infection. Mock consists of empty pCAMBIA1302 vector infiltrated leaves. Input is immunoblot (IB) with anti-GFP antibody of crude protein extracts. Ponceau staining represents equal loading of immunoprecipitated (IP) samples.

To explore involvement of MAPKs in pathogen signaling in response to *X. oryzae* infection in rice, the transcript level of the selected members (group A, B and C) of the gene family was studied. 15 days old rice seedlings were treated with *X. oryzae* suspension for different time period. The transcript level of OsMPK7 was rapidly increased along with the transcripts of OsMPK3, OsMPK4 and OsMPK6 within four hours of *X. oryzae* treatment (Fig. 1a).

Hydrogen peroxide (H_2_O_2_) and salicylic acid (SA) are important messengers that trigger MAPK signaling cascades in response to pathogens^12^,^13^. Hence, effect of H_2_O_2_ and SA on OsMPK7 transcript accumulation was assessed. The transcript level of OsMPK7 was substantially increased within two hours of H_2_O_2_ treatment, whereas SA treatment had no significant effect on OsMPK7 transcript level (Fig. 1a). These results illustrated that *OsMPK7* was up-regulated in response to *X. oryzae* and H_2_O_2_ treatment but not by SA treatment, suggesting its possible role in early PAMP triggered immune response against *X. oryzae* pathogen. The other MAPK members, *OsMPK3*, *OsMPK4* and *OsMPK6* showed upregulation under both, H_2_O_2_ and SA treatments.

To further examine possible role of OsMPK7 in rice- *X. oryzae* interplay, OsMPK7-GFP fusion was expressed in 50 days old rice leaves. The transformation and expression of OsMPK7 gene in rice leaves was confirmed by quantitative RT-PCR and semi quantitative RT-PCR (Fig. S1a and S1c). In parallel the leaves transformed with empty vector served as control. After infection of transformed leaves with *X. oryzae*, OsMPK7 protein was immunoprecipitated using anti-GFP antibody and immunoblotting was carried out with pTEpY antibody^23^. Increase in phosphorylation of OsMPK7 was observed in the samples infected with *X. oryzae* as compared to non-infected controls (Fig. 1b). Immunoblot (IB) with anti-GFP antibody suggests the presence of OsMPK7-GFP protein. The observation illustrated *X. oryzae* mediated activation of OsMPK7 and its possible role during *X. oryzae* infection.

### OsMPK7 imparts resistance towards *X. oryzae* infection

**Figure 2:**
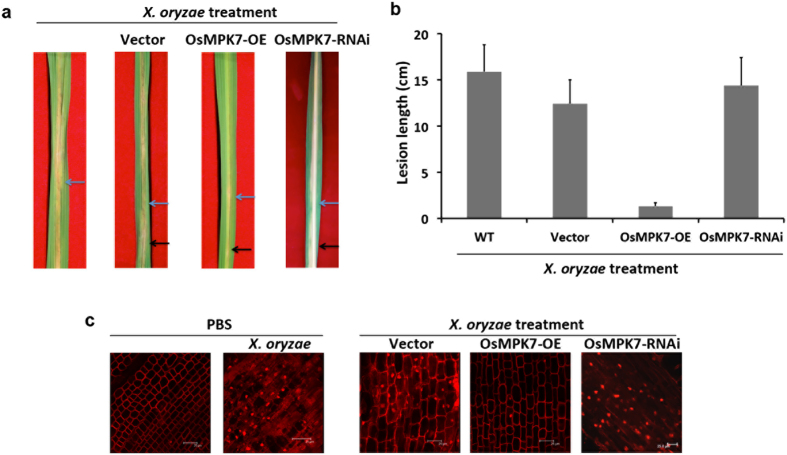
OsMPK7 induces resistance towards *X. oryzae* infection. (**a**) Rice resistance assay in leaves. 50 days old rice leaves with overexpressed and silenced OsMPK7 gene expression were infected with *X. oryzae* pathogen. As control, empty vector infiltrated leaves were used. Disease lesions were formed in control leaves and leaves with silenced OsMPK7 gene expression, in contrast, leaves overexpressing OsMPK7 prior to *X. oryzae* treatment showed reduced disease lesions. Blue arrow indicates point of *X. oryzae* inoculation and black arrow indicates point of *Agrobacterium* infiltration. Rice leaves were transformed with the respective constructs 24 h prior to the infection with *X. oryzae*. (**b**) Quantification of disease lesions formed after *X. oryzae* infection in rice leaves with overexpressed and silenced *OsMPK7*. Lesion length was measured 10 days after infection in 7–8 different leaves and similar results were obtained in four independent experiments. Error bar indicates standard deviation of readings from four independent experiments. (**c**) Cell death assay in rice roots. One week old rice roots with overexpressed and silenced *OsMPK7* were infected with *X. oryzae*. Roots treated with PBS were used as control. OsMPK7 overexpressed roots showed less cell death upon *X. oryzae* infection, whereas control roots and roots with silenced *OsMPK7* showed more cell death and nuclear fragmentation. Scale: 25 μm. The experiment was repeated independently four times wherein similar results were obtained.

*X. oryzae* produces disease lesions on rice leaves, a prominent symptom observed during infection^25^,^26^. To understand the role of OsMPK7 in *X. oryzae* infection, rice resistance assay was carried out in rice leaves with overexpressed and silenced OsMPK7 gene expression raised by transient transformation (modified as described in ref. 23). Leaves transformed with empty vector were used as control. These leaves were infected with *X. oryzae* and observed for disease lesion formation. The transformation and expression of OsMPK7 gene in rice leaves was confirmed by quantitative RT-PCR and semi quantitative RT-PCR (Fig S1a, S1b, S1c and S1d). Transformation efficiency in rice leaves was measured by calculating percentage of transformed leaves and was found to be 82% for OsMPK7 overexpressed leaves and 79% for silenced leaves (Fig. S1e). At 10 days after infection (DAI), leaves overexpressing OsMPK7 prior to *X. oryzae* infection exhibited shorter disease lesions (0.2 cm) in contrast to the leaves with silenced OsMPK7 expression (14 cm) and vector control leaves (12 cm) (Fig. 2a,b and Table 1). This suggested that over-expression of OsMPK7 prevents formation of disease symptoms by *X. oryzae* infection and that OsMPK7 is an important signaling component mediating cues necessary for imparting disease resistance.

*X. oryzae* being biotrophic microorganism suppresses host immune system and feeds on it. The secretion of cell wall degrading enzymes and extensive feeding by the pathogen on host cells causes nutrient depletion leading to cell death^11^,^26^. Extent of cell death caused in *X. oryzae* infection was further assessed in one week old rice roots after incubating them in *X. oryzae* suspension. The roots treated with *X. oryzae* showed extensive cell death as compared to control roots (Fig. 2c). Based on this observation the role of OsMPK7 in reducing cell death was explored in rice roots with overexpressed and silenced OsMPK7 gene expression, after *X. oryzae* infection. The transformation and expression of OsMPK7 gene in rice roots was confirmed by quantitative RT-PCR and semi quantitative RT-PCR (Fig. S1f and S1g). It was observed that roots overexpressing OsMPK7 showed less cell death as compared to the vector control and OsMPK7 silenced roots (Fig. 2c). This result implied the involvement of OsMPK7 in imparting resistance against *X. oryzae* infection.

### OsMKK3 is induced by *X. oryzae* infection and is upstream kinase activating OsMPK7

**Figure 3:**
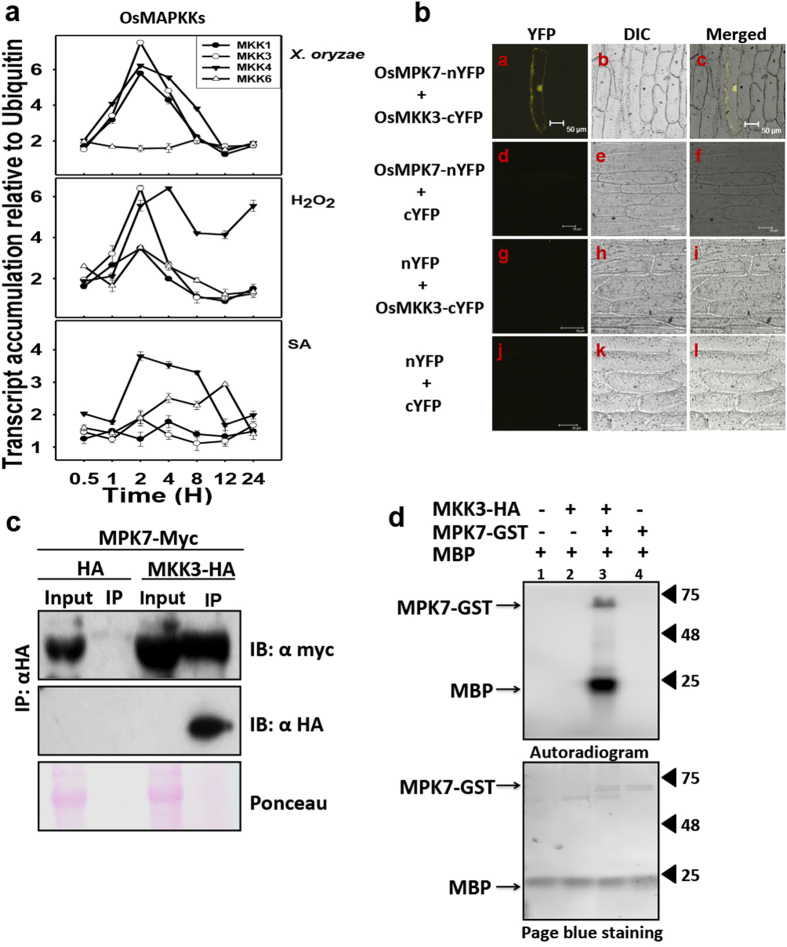
OsMKK3 interacts and activates OsMPK7. (**a**) Transcript levels of MAPKK genes were examined in 15 days old rice seedlings in response to *X. oryzae*, H_2_O_2_ and Salicylic acid (SA) treatment. Ubiquitin and actin gene was considered as internal control. The experiments were repeated three times with three technical replicates. (**b**) Bimolecular fluorescence assay (BiFc) performed using OsMPK7-eYFP_N173_ and OsMKK3-eYFP_C155_ fusion protein reveals interaction between OsMPK7 and OsMKK3. YFP fluorescence was observed in onion epidermal cells expressing both OsMKK3 and OsMPK7 fusion proteins as seen in (**a**) Epifluorescence, (**b**) bright field and (**c**) merged images of onion epidermal cells. No YFP fluorescence is observed in negative controls (**d**,**g** and **j**). Scale bar: 50 μm. (**c**) Immunodetection of complex formation of OsMPK7-Myc and OsMKK3-HA *in planta* using Co- immunoprecipitaion assay (CoIP). OsMPK7-Myc was co-immunoprecipitated with OsMKK3-HA using anti-HA antibody from plant protein extracts of tobacco leaves transiently expressing both OsMPK7-Myc and OsMKK3-HA. Immunoblotting was carried out with anti-Myc antibody. No band corresponding OsMPK7-Myc is seen in control sample. The lower panel shows the presence of OsMKK3-HA. (**d**) *In vitro* kinase assay showing phosphorylation of OsMPK7 by its upstream kinase, OsMKK3. OsMKK3-HA protein was immunoprecipitated from plant protein extract of tobacco leaves transiently expressing OsMKK3-HA and phosphorylated OsMPK7-GST, which in-turn phosphorylated MBP. Phosphorylation signal of OsMPK7 and MBP was detected by autoradiography.

To get an insight into the upstream kinase activating OsMPK7 in response to *X. oryzae* infection, the transcript level of OsMAPKKs was analyzed in *X. oryzae*, H_2_O_2_ and SA treatments. It was found that out of eight members *OsMKK1*, *OsMKK3* and *OsMKK4* were up-regulated in *X. oryzae* and H_2_O_2_ treatment (Fig. 3a) and only *OsMKK4* and *OsMKK6* showed relative upregulation in SA treatment (Fig. 3a). This observation indicated possible role of OsMKK1, OsMKK3 and OsMKK4 in *X. oryzae* mediated signaling and could act as upstream kinase for OsMPK7. To reveal the upstream kinase that interacts and activates OsMPK7 during *X. oryzae* infection, *in planta* interaction studies were performed. We speculated the involvement of OsMKK3 as an upstream kinase of OsMPK7 since yeast two hybrid study performed in our group previously had shown interaction of OsMPK7 with OsMKK3^8^. To investigate this hypothesis, *in planta* interaction of OsMPK7 and OsMKK3 was studied using Bimolecular Fluorescence complementation (BiFc) assay and co-immunoprecitipation (Co-IP) assay. For BiFc, OsMPK7-eYFP_N173_ and OsMKK3-eYFP_C155_ fusion proteins were co-expressed in onion epidermis by particle bombardment method. After 72 hours of incubation onion epidermis was observed under confocal microscope for YFP fluorescence. YFP fluorescence was observed in epidermal cells expressing both OsMPK7-eYFP_N173_ and OsMKK3-eYFP_C155_ whereas no fluorescence was observed in vector control samples (Fig. 3b). The observation demonstrated *in planta* interaction of OsMPK7 with OsMKK3. This interaction was further validated using Co-IP assay wherein immunoprecipitation was carried out using the protein extracts from *N. benthamiana* leaves expressing OsMKK3-HA and OsMPK7-Myc tags using anti-HA antibody. The immunoprecipitated samples were subsequently subjected to immunoblotting using anti-Myc antibody. Signal corresponding to OsMPK7-Myc was detected in anti-HA immunoprecipitated samples expressing both OsMPK7-Myc and OsMKK3-HA. No signal corresponding to OsMPK7-Myc was observed in anti-HA immunoprecipitated sample expressing only OsMPK7-Myc (Fig. 3c). This confirmed the interaction of OsMPK7 with OsMKK3 *in planta* and also suggested that OsMPK7 might work downstream of OsMKK3 in *X. oryzae* mediated signaling.

For OsMPK7 to bestow its function has to get activated through phosphorylation by an upstream MAPKK. To analyse this, *in vitro* phosphorylation assay (kinase assay) was carried out using bacterially expressed OsMPK7-GST and plant expressed OsMKK3-HA protein isolated from infected rice leaves. Interestingly the outcome revealed that OsMPK7 was indeed phosphorylated and activated by OsMKK3, which in turn phosphorylated MBP (myelin basic protein), a universal MAPK substrate (Fig. 3d). This observation confirmed the interaction and activation of OsMPK7 by OsMKK3 through phosphorylation, and suggested OsMKK3 to be an upstream kinase of OsMPK7 probably working in *X. oryzae* mediated signaling.

### OsMKK3-OsMPK7 module is involved in rice –*X. oryzae* interplay

**Figure 4:**
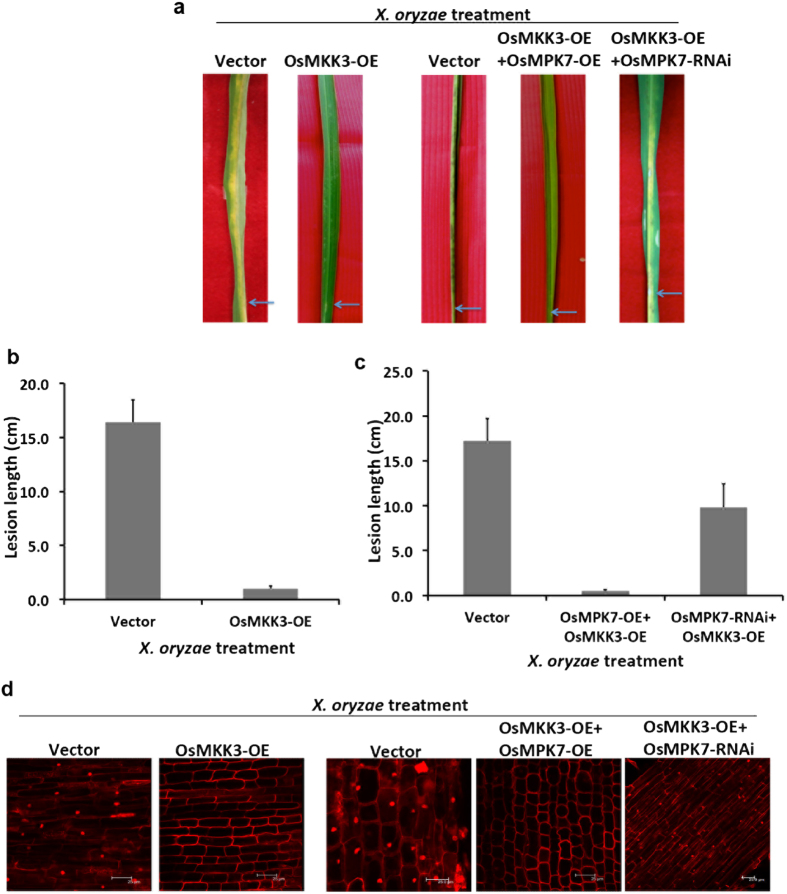
OsMKK3-OsMPK7 module imparts resistance to *X. oryzae* infection. (**a**) Rice resistance assay in rice leaves. 50 days old rice leaves transformed with empty vectors prior to *X. oryzae* infection showed disease lesions similar to leaves with overexpressed OsMKK3 and silenced OsMPK7 gene expression. Whereas, leaves overexpressing OsMKK3 alone or together with its downstream kinase OsMPK7 prior to *X. oryzae* infection showed less disease lesions formation. Blue arrow indicates point of *X. oryzae* inoculation. (**b**) and (**c**) Quantification of disease lesions formed after *X. oryzae* infection in rice leaves overexpressing OsMKK3 alone or together with overexpressed and silenced *OsMPK7*. Empty vector transformed leaves were taken as control. Lesion length was measured 10 days after infection in 6–7 different leaves and similar results were obtained in four independent experiments. Error bar indicates standard deviation of readings from four independent experiments. (**d**) Cell death assay in rice roots overexpressing OsMKK3 alone and together with OsMPK7 prior to *X. oryzae* infection showed less cell death in contrast to control roots and roots with overexpressed OsMKK3 and silenced OsMPK7. Scale: 25 μm. Experiment was repeated four different times wherein similar results were obtained.

After establishing that OsMKK3 is upstream kinase of OsMPK7, we speculated its role in rice-*X. oryzae* interaction. To prove this, rice resistance assay was carried out in 50 days old rice leaves by overexpressing OsMKK3 alone and in combination with OsMPK7. This assay was also performed with overexpressed OsMKK3 and silenced OsMPK7 gene expression. The transcript level of OsMKK3 and OsMPK7 in these transformed rice leaves was confirmed by quantitative RT-PCR (Fig. S2a, S2b and S2c), semi-quantitative RT-PCR (Fig. S2d, S2e and S2f) and western blotting (Fig. S2k). Transformation efficiency was measured by calculating percentage of transformed leaves and was found to be 72% for OsMKK3 overexpressed leaves, 61% for OsMKK3 + OsMPK7 overexpressed leaves and 69% for OsMKK3 overexpressed +MPK7 silenced leaves (Fig. S2g). After 24 hours these rice leaves were infected with *X. oryzae* pathogen and formation of disease lesions was observed at 10 DAI. Interestingly, it was observed that leaves overexpressing either OsMKK3 alone or OsMKK3 along with OsMPK7 showed very short disease lesions of 0.2 cm in length (Fig. 4a,b,c and Table 1). In contrast leaves with overexpressed OsMKK3 and silenced OsMPK7 showed extensive formation of disease lesions with 10 cm length (Fig. 4a,b,c and Table 1). This observation indicated that OsMPK7 and its upstream kinase OsMKK3 works concurrently in imparting resistance to the formation of disease symptoms in *X. oryzae* infection. To further get insight into this phenomenon, cell death assay in rice roots was carried out in which OsMKK3 alone and along with OsMPK7 were overexpressed in one week old rice roots. The roots with overexpressed OsMKK3 and silenced OsMPK7 were also used in the experiment. Transcript accumulation of OsMPK7 and OsMKK3 in rice roots was confirmed by Q-RT PCR (Fig. S2h and S2i), semi Q-RT PCR and western blotting (Fig. S2j and S2k). After infection with *X. oryzae* it was observed that roots expressing either of OsMKK3 or OsMKK3 with OsMPK7 showed very less cell death (Fig. 4d). Whereas more cell death and nuclear fragmentation was observed in vector control roots and in roots with overexpressed OsMKK3 and silenced OsMPK7 (Fig. 4d). This showed that OsMKK3 along with OsMPK7 positively regulates defence response against rice bacterial pathogen *X. oryzae*.

### OsMPK7 interacts with OsWRKY30 *in planta*

**Figure 5:**
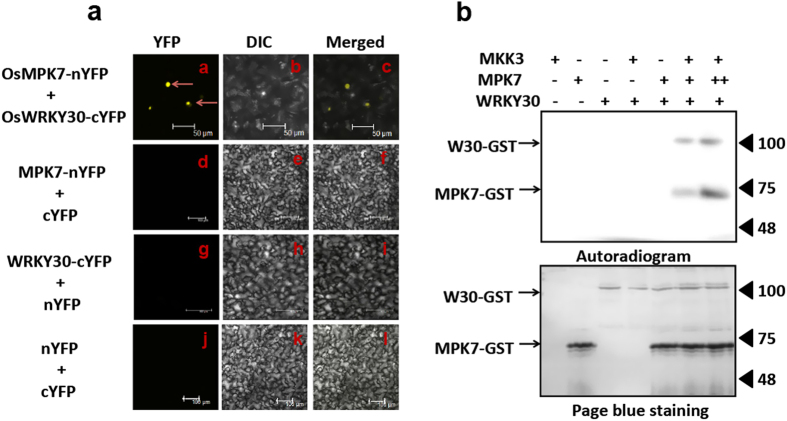
OsMPK7 interacts and phosphorylates downstream OsWRKY30. (**a**) Bimolecular fluorescence assay (BiFc) performed using OsMPK7-eYFP_N173_ and OsWRKY30-eYFP_C155_ fusion protein reveals interaction between OsMPK7 and OsWRKY30. YFP fluorescence was observed in *N. benthamiana* cells expressing both OsWRKY30 and OsMPK7 fusion proteins as seen in (**a**) Epifluorescence, (**b**) bright field and (**c**) merged images. No YFP fluorescence is observed in negative controls (**d**,**g** and **j**). Scale bar: 50 μm. (**b**) *In vitro* kinase assay showing phosphorylation of OsWRKY30 by its upstream interacting kinase, OsMPK7. OsMPK7 phosphorylated OsWRKY30 in presence of plant expressed OsMKK3-HA protein however bacterially expressed OsMPK7 alone did not phosphorylate OsWRKY30. “+*” Indicate double concentration of OsMPK7. Phosphorylation signal of OsMPK7 and MBP was detected by autoradiography.

To get further insight into OsMKK3-OsMPK7 module working in *X. oryzae* infection, we sought to decipher the downstream target involved in of OsMKK3-OsMPK7 module. We started with an *in silico* approach and used STRING9.0 database for possible screening protein-protein interaction. Out of various probable interacting partners of OsMPK7 OsWRKY30 was identified as one of them (Table S2), which was also speculated to be an interacting partner of OsMPK7 by yeast two hybrid analysis^27^. OsWRKY30 is a member of WRKY family transcription factor in rice that plays important role in plant defence mechanism^28^. To analyse the participation of OsWRKY30 in OsMKK3-OsMPK7 module working against *X. oryzae*, interaction of OsWRKY30 with OsMPK7 was first confirmed *in-planta* through BiFc assay. YFP fluorescence was observed in the nucleus of cells co-expressing OsMPK7-eYFP_N173_ and OsWRKY30-eYFP_C155_, however no fluorescence was observed in leaves infiltrated with control vectors (Fig. 5a) which suggested that OsMPK7 and OsWRKY30 interacted in the nucleus.

Since OsMPK7 interacted with OsWRKY30, we presumed that it might be the upstream kinase phosphorylating OsWRKY30. To prove this, *in vitro* kinase assay was carried out using bacterially expressed OsMPK7 and OsWRKY30 protein. It was observed that bacterially expressed OsMPK7 alone did not phosphorylate OsWRKY30. However, when OsMPK7 was incubated with post-infected plant protein extract expressing OsMKK3, it phosphorylated OsWRKY30 (Fig. 5b). The observation suggested that OsMKK3 activated OsMPK7 which subsequently phosphorylated and activated OsWRKY30. The observation further indicated that OsWRKY30 might act downstream of OsMPK7 in OsMKK3-OsMPK7 pathway working during *X. oryzae* infection in rice.

### OsWRKY30 acts downstream of OsMKK3-OsMPK7 module in *X. oryzae* mediated signaling

**Figure 6:**
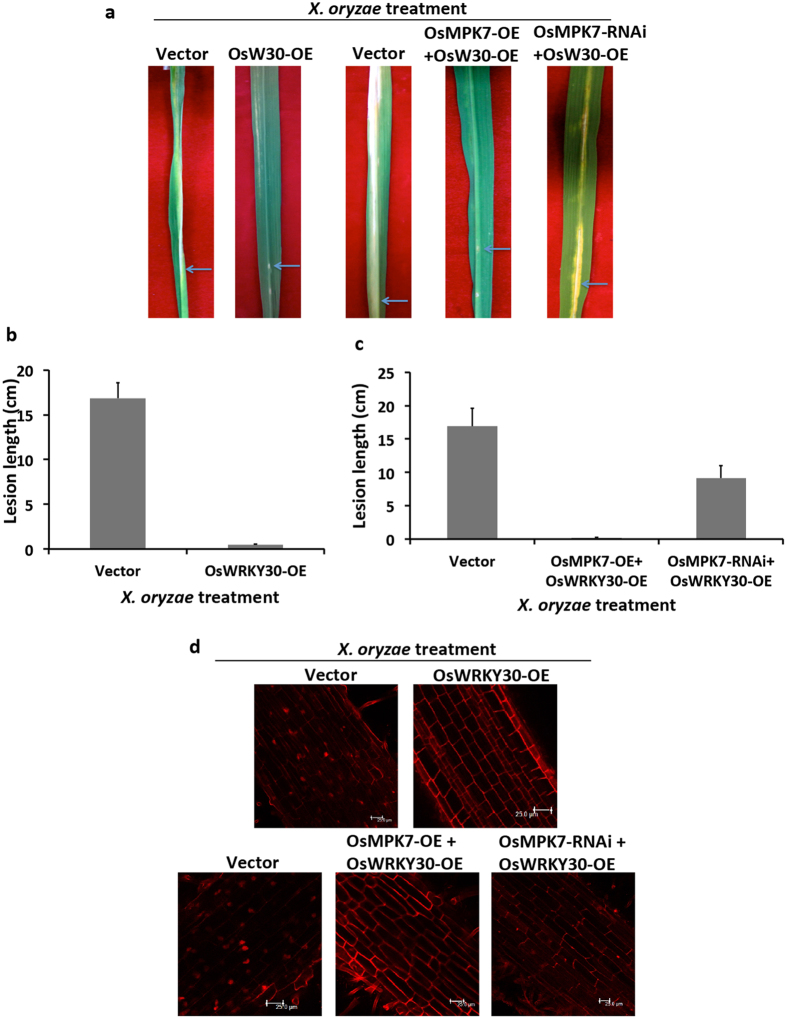
OsMPK7-OsWRKY30 signaling module mediates disease resistance against *X. oryzae* infection. (**a**) Rice resistance assay in rice leaves. 50 days old rice leaves overexpressing OsWRKY30 alone or together with its upstream kinase OsMPK7 prior to *X. oryzae* infection showed less disease lesions formation. However, control leaves with empty vectors and leaves with overexpressed OsWRKY30 and silenced OsMPK7 gene expression showed more disease symptoms. Blue arrow indicates point of *X. oryzae* inoculation. (**b**) and (**c**) Quantification of disease lesions formed after *X. oryzae* infection in rice leaves overexpressing OsWRKY30 alone or together with overexpressed and silenced *OsMPK7*. Empty vector transformed leaves were taken as control. Lesion length was measured 10 days after infection in 7–8 different leaves and similar results were obtained in four independent experiments. Error bar indicates standard deviation of readings from four independent experiments. (**d**) Cell death assay in rice roots overexpressing OsWRKY30 alone and together with OsMPK7 prior to *X. oryzae* infection showed less cell death in contrast to roots with overexpressed OsWRKY30 and silenced OsMPK7. Control roots transformed with empty vectors also showed more cell death in *X. oryzae* infection. Scale: 25 μm. Experiment was repeated four different times wherein similar results were obtained.

The role of OsWRKY30 was tested in defence response against *X. oryzae*, mediated by OsMKK3-OsMPK7 pathway by performing rice resistance assay and cell death assay in rice leaves and roots, respectively. For rice resistance assay, OsWRKY30 alone and OsWRKY30 together with OsMPK7 were overexpressed in rice leaves. To specifically delineate the function of OsMPK7-OsWRKY30 module, OsWRKY30 was also overexpressed along with silenced *OsMPK7*. The transcript level of OsWRKY30 and OsMPK7 in these transformed rice leaves was confirmed by quantitative RT-PCR and semi-quantitative RT-PCR (Fig. S3a–S3f). Transformation efficiency was found to be 84% for OsWRKY30 overexpressed leaves, 64% for OsWRKY30 + OsMPK7 overexpressed leaves and 65% for OsWRKY30 overexpressed +OsMPK7 silenced leaves (Fig. S3g). It was observed that leaves overexpressing either OsWRKY30 alone or together with OsMPK7 showed very less disease lesion formation after *X. oryzae* infection, with lesion length of less than 0.5 cm. While the leaves with overexpressed OsWRKY30 and silenced OsMPK7 showed more disease lesion formation with lesion length of 10 cm (Fig. 6a,b,c and Table 1). This demonstrated the involvement of OsWRKY30 in *X. oryzae* mediated signaling in rice.

To confirm this further cell death assay was carried out in which OsWRKY30 alone and together with OsMPK7 was overexpressed in rice roots. Apart from it, roots with overexpressed OsWRKY30 and silenced OsMPK7 gene expression were also used in this assay. Transcript accumulation of OsMPK7 and OsWRKY30 in rice roots was confirmed by Q-RT PCR and semi Q-RT PCR (Fig. S3h–S3k). The result revealed that roots overexpressing either OsWRKY30 alone or together with OsMPK7 showed very less cell death, as compared to the control roots or roots with overexpressed OsWRKY30 and silenced OsMPK7 (Fig. 6d). Both the outcomes confirmed the involvement of OsWRKY30 in providing disease resistance and placed OsWRKY30 downstream of OsMPK7 in OsMKK3-OsMPK7 pathway in *X. oryzae* infection.

### OsMKK3-OsMPK7 module induces defence response genes and restricts the migration of pathogen

**Figure 7:**
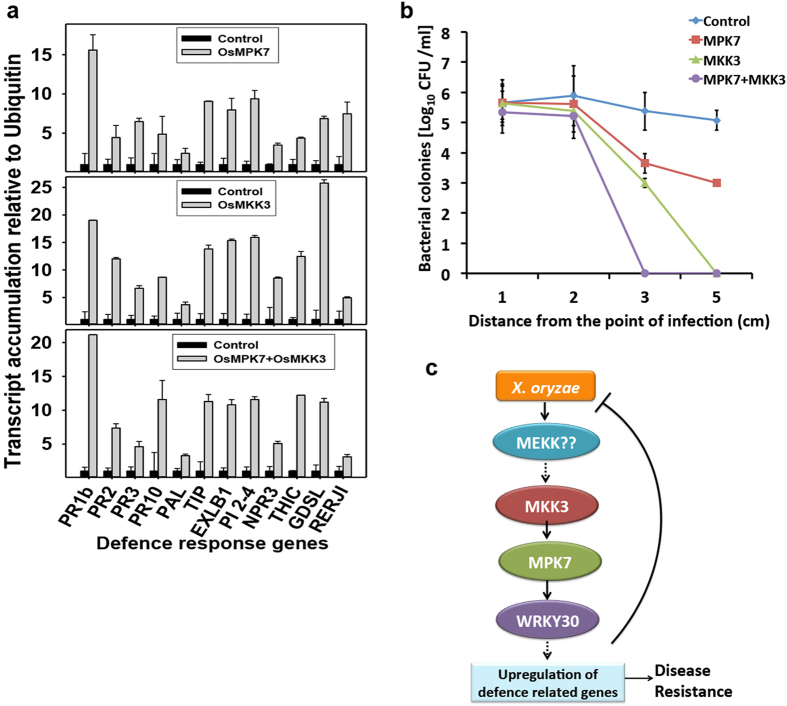
OsMKK3-OsMPK7 regulates expression of defence response genes and restricts migration of *X. oryzae*. (**a**) Quantification of transcript levels of PR genes and defence genes involved in maintenance of cell structure and metabolism in rice leaves overexpressing OsMPK7, OsMKK3 and OsMKK3-OsMPK7 module by Q-RT-PCR. Leaves transformed with empty vectors were used as controls. Defence response genes analysed were: Pathogenesis related genes (PR1b, PR2, PR3, PR10), Phenylalanine ammonia lyase (PAL), Tonoplast membrane integral protein (TIP), Expansin like B1 precursor (EXLB1), Pathogen induced protein 2–4 (PI 2–4), Ankyrin repeat protein (NPR3), Thiamine biosynthesis protein (ThiC), GDSL lipase like protein (GDSL), Helix-loop-helix containing transcription factor (RERJI). Ubiquitin gene was used as internal control. Gene expression analysis was carried out independently three times. (**b**) Bacterial colony count assay in rice leaves overexpressing OsMKK3-OsMPK7 module. Rice leaves (50 days old) overexpressing OsMPK7, OsMKK3 and OsMKK3-OsMPK7 module restricted the migration of *X. oryzae* as seen by decrease in colony counts observed in adjacent area of infection. (**c**) Proposed model for MAPK signaling cascade depicting involvement of OsMKK3-OsMPK7-OsWRKY30 module in *X. oryzae* infection. Upon perception of *X. oryzae* related cues by upstream receptor causes activation of this myriad MAPK cascade in which activated OsMKK3 phosphorylates and activate OsMPK7 which then further activate OsWRKY30 by phosphorylation. This activated OsWRKY30 then triggers regulation of defence response gene expression and ultimately imparting resistance towards the pathogen.

Recognition of pathogen by plant triggers several early and late defence responses. One of the early responses includes activation of MAPKs and one of the late responses includes activation of wide array of defence response genes^9^,^10^. It was hypothesized that OsMKK3-OsMPK7 module imparts resistance to the *X. oryzae* infection by activating several downstream defence response genes. For this several defence response genes regulated in *X. oryzae* infection were selected^29^ and their transcript analysis was carried out in leaves overexpressing OsMPK7, OsMKK3 and OsMKK3-OsMPK7. It was observed that most of the genes were preferentially up-regulated in these tissues (Fig. 7a). The genes which showed up-regulation were mostly Pathogenesis-Related (PR) genes encoding PR proteins and genes involved in maintenance of cell structure and metabolism. The result suggested that OsMKK3-OsMPK7 module induces resistance towards *X. oryzae* by regulating expression of genes involved in defence response and genes involved in cell structure and metabolism. Thus, altering the cell structure and making plants resistance against the bacterial attack.

One of the defence response induced upon pathogen attack in PTI is strengthening of cell wall which prevents the entry of pathogen and further infection to surrounding area^10^,^30^. Emphasizing on previous observation that OsMKK3-OsMPK7 module regulates expression of genes involved in cell structure and metabolism, it was postulated that this module alter the cell morphology upon pathogen attack thus restricting entry of pathogen in adjacent cells. To decipher this idea, the movement of pathogen surrounding the infected area was studied by bacterial colony count. The bacterial colonies were counted in each of 1 cm leaf segments overexpressing OsMKK3-OsMPK7 module, starting from point of infection. The colony count was reduced drastically in leaf segments away from the point of infection overexpressing OsMPK7, OsMKK3 and OsMKK3-OsMPK7 module in-contrast to control leaves (Fig. 7b). The observation further illustrated the possible mechanism of resistance conferred by OsMKK3-OsMPK7 module against *X. oryzae*.

Based on our findings we propose a simple model of involvement of OsMKK3-OsMPK7-OsWRKY30 module in imparting resistance against *X. oryzae* infection in rice by inducing several defence related genes and genes involved in cell wall metabolism and cell structure maintenance, thereby restricting the migration of bacteria along the leaf surface (Fig. 7c).

## Discussion

Mitogen activated protein kinase cascade has been implicated in several developmental, biotic and abiotic cues. However, the functional information about different members of MAPK in rice is still very limited. On top of this, the group C members is least explored. In the current study we provide evidence of a group C member of MAPK, OsMPK7 during *Xanthomonas oryzae*-rice interaction. We propose a OsMKK3-OsMPK7-OsWRKY30 module working during *X. oryzae* mediated signaling pathway in rice. *X. oryzae* infection causes OsMPK7 phosphorylation by OsMKK3 which in-turn phosphorylate OsWRKY30, thereby mediating defense responses. Furthermore, this module results in induction of defence related genes and restricts the migration of the pathogen thereby imparting disease resistance.

In rice OsMAPK4 (alternative name for OsMPK7) was studied to be transcriptionally regulated by developmental and different environmental stages^31^ and in circadian rhythm^24^. Our initial observation of transcriptional regulation of OsMPK7 upon *X. oryzae* infection led us to investigate the role of OsMPK7 in transmitting *X. oryzae* related cues. Upon perception of PAMP/MAMP by upstream receptor, several early and late immune responses gets initiated including activation of MAPKs^10^,^15^,^16^,^17^. The fact that the transcript of OsMPK7 responded to only H_2_O_2_ treatment and not to SA treatment made us to explore the role of OsMPK7 in detail during *X. oryzae*-rice interaction. Since the other three MAPKs (OsMPK3, OsMPK4 and OsMPK6) also responded to *X. oryzae*, H_2_O_2_ and SA treatments we do not rule out the possibility of their involvement in pathogen signaling. H_2_O_2_ is known to activate several of the MAPKs, like MPK3, MPK4 and MPK6 in Arabidopsis, MPK1/2 in tomato and WIPK and SIPK in *Nicotiana* species^12^,^14^,^32^. SA produced in ETI is known to play role in regulating several MAPKs^33^,^34^. Not responding to SA treatment indicated the involvement of OsMPK7 in PAMP triggered immunity. Immunokinase assay using pTEpY antibody which specifically binds to phosphorylated TEY domain^35^,^36^ indicated specifc phoshorylation of OsMPK7 after infection with *X. oryzae*. This observation further supported our speculation of involvement of OsMPK7 in pathogen signaling mediated by *X. oryzae*.

The virulence of *X. oryzae* is mainly provoked by type II and type III secretion systems which secrets cell wall degrading enzymes allowing pathogen to feed on the living plant tissue ultimately causing disease lesions on the leaves^11^,^25^,^26^. To ascertain the role of OsMPK7 during *X. oryzae* infection OsMPK7 was either transiently overexpressed or silenced in the rice leaves and roots. Overexpression of MAPKs is known to induce defence responses, either imparting resistance or susceptibility against disease symptoms. OsMPK6 expression and activation engendered local resistance to *X. oryzae* infection by triggering hypersensitive reactions^37^. Interestingly, it was observed that rice leaves overexpressing OsMPK7 prior to *X. oryzae* infection developed smaller disease lesions as compared to the OsMPK7 silenced leaves. Similarly, rice roots overexpressing OsMPK7 showed less cell death, however opposite effect was observed in *OsMPK7* silenced roots. This exemplified the participation of OsMPK7 positively in inducing defence responses against *X. oryzae* infection in rice.

We further identified upstream kinase and also downstream target of OsMPK7 possibly playing role in the same pathway. Using an *in silico* docking analysis followed by yeast two hybrid analysis we had reported earlier interaction of OsMKK3 with OsMPK7^8^. Preliminary study in Arabidopsis reported MKK3 to interact with MPK7 that participated in pathogen signaling against *P. Syringae*^18^. In the present study we observed upregulation of the transcript of OsMKK3 along with OsMKK1 and OsMKK4 by *X. oryzae* and H_2_O_2_ treatment. Considering this background we authenticated the interaction of OsMKK3 and OsMPK7 *in planta* by BiFc and CoIP assay. Furthermore *in vitro* phosphorylation assay confirmed the phosphorylation of OsMPK7 by OsMKK3 and indicated OsMPK7 to be a direct substrate of OsMKK3.

Effect of any MAPK depends on its upstream activating MAPK cascade components. Same MAPKs are known to be activated by several different upstream kinases while the response set by a single pathway becomes very specific. Though MAPK cascade positively regulates defence response, they are also known to act as negative repressor in response to pathogen attack and this mostly depends on the function of upstream components. OsMPK3 and OsMPK6 are known to be activated by both OsMKK4 and OsMKK6. OsMKK4 regulates OsMPK3/6 to inhibit PR gene expression while their activation through OsMKK6 causes positive defence response^21^,^38^. The most deliberately studied MPK4 is known to negatively regulate immune response and is itself regulated by MEKK1-MKK1/2^39^. Since OsMKK3 was revealed as an upstream kinase of OsMPK7 it was necessary to study the role of OsMKK3 alone and together with OsMPK7 during *X. oryzae* infection. Rice resistance and cell death assay illustrated that rice leaves and roots overexpressing either OsMKK3 alone or OsMKK3-OsMPK7 module did not developed any disease symptoms caused by *X. oryzae* infection. However, rice tissues with overexpressed *OsMKK3* and silenced *OsMPK7* expression showed more disease symptoms. The observation indicated involvement of OsMKK3 in positively regulating OsMPK7 in imparting disease resistance against *X. oryzae* infection.

Besides the role of an upstream MAPK in signaling cascade, involvement of downstream MAPK substrate is equally required to pass on the signal ultimately generating a response. In order to find interacting proteins of OsMPK7 several protein-protein interaction techniques were employed from which OsWRKY30 was found to be interacting with OsMPK7. The interaction of OsMPK7 with OsWRKY30 was confirmed using BiFc assay while *in vitro* phosphorylation assay established it to be a phosphorylation target of OsMPK7. Since OsWRKY30 was confirmed as downstream substrate of OsMPK7, it was mandatory to investigate its function in mediating *X. oryzae* related cues. WRKY transcription factors in rice are known to be induced upon pathogen attack and execute expression of defence related genes^28^. This is generally due to the presence of “WRKY” DNA binding domain in WRKY transcription factors that binds to “W box” in promoter of defence related genes. WRKY factors are known to act both positively and negatively leading to either activation or suppression of gene expression^28^. Rice resistance assay and cell death assay performed in rice leaves and roots overexpressing OsWRKY30 alone and together with OsMPK7 exhibited very less disease symptoms. On the other hand, leaves and roots overexpressing OsWRKY30 and silenced OsMPK7 showed more disease symptoms. These observations presented a positive involvement of OsWRKY30 in transmitting *X. oryzae* related signals from OsMKK3-OsMPK7 module thereby generating immune response.

To get insight into the mechanism by which OsMKK3-OsMPK7 module imparts resistance towards *X. oryzae* infection we studied the expression of known defence related genes. Many of the MAPKs have been identified to induce basal defence responses like activation of defence response genes, ROS burst, cell wall strengthening, phytoalexin biosynthesis, etc^12^,^21^,^37^. Some of the defence response genes^29^ were analyzed and found to be preferentially up-regulated in tissues overexpressing OsMPK7 and OsMKK3. Among this, the defence related genes which got induced involved *TIP*, *EXLB1*, *PI 2–4*, *NPR3*, *THIC* and *GDSL*. These genes were reported to be induced upon treatment of secreted cellulase of *X. oryzae*, which is also known to induce innate immune response against *X. oryzae*^29^. Also these up-regulated genes are mainly involved in maintaining cell structure and metabolism which points to the fact that the OsMKK3-OsMPK7 module mediates changes in cell structure and metabolism. These changes might be necessary for induction of immunity towards pathogen attack. Apart from these genes some of the PR genes like *PR1b*, *PR2* and *PR10* were also found to be upregulated in OsMPK7 and OsMKK3 expressing tissues. It is generally noted that expression of PR genes is correlated with resistance to the disease against pathogen^38^. Upregulation of these defence related genes and genes involved in cell structure maintenance by OsMKK3-OsMPK7 module could be the reason of disease resistance mediated by this pathway.

The induction of defence response genes involved in controlling cell structure and metabolism indicated that OsMKK3-OsMPK7 module positively induces defence against *X. oryzae* by modulating plant cell wall. This might result in restricted entry and growth of pathogen in plant cell. In the present study, the bacterial count assay was carried out to explain the mechanism of resistance provided by OsMKK3-OsMKP7 module against *X. oryzae*. It was revealed that OsMKK3-OsMPK7 restricts the migration and growth of pathogen in surrounding leaf area from the point of infection and thus imparting resistance towards *X. oryzae* infection.

In summary, the results presented in this article suggest the role of a least studied group C rice MAPK, OsMPK7 in pathogen signaling. This study reveals the positive involvement of OsMPK7 in imparting disease resistance against *X. oryzae*. Furthermore, an important role of OsMKK3 in regulating function of OsMPK7 is explained. Additionally, OsWRKY30 is revealed to be downstream target of OsMPK7 mediating defence related signals from OsMPK7. Moreover, this work further provides the mechanism of action of OsMKK3-OsMPK7-OsWRKY30 module in regulating disease resistance against *X. oryzae*.

## Materials and Methods

### Plant growth conditions and treatments

For H_2_O_2_, SA, *X. oryzae* treatments, rice (*Oryza sativa* L. *indica* cultivar) plants were grown in growth chamber (SCILAB instrument, Taiwan) at 30 °C with 16 hours light and eight hours dark cycles. 15 days seedlings were treated with 20 mM H_2_O_2,_ 20 μM SA and *X. oryzae* (O.D. 0.8) for different time periods. For rice resistance assay, rice plants were grown in phytotron chamber (Conviron, US/Canada) with growth conditions of temperature 30 °C, 16 hours light (250 μE/m^2^/s) and eight hours dark and with 70% humidity.

### Expression and purification of recombinant proteins

For bacterially expressed recombinant proteins, the cloned constructs in pGEX4T-2 (GE Healthcare, UK) vector were used. The protein was induced with 1 mM isopropyl β–D-1 thiogalactopyranoside (IPTG) in *E. coli* BL21 transformed with pGEX4T-2 constructs. Fusion protein was purified using glutathione sepharose beads (GE Healthcare, UK).

For *in planta* recombinant proteins, binary vector constructs in pSPYNE(R)173 and pSPYCE(M) were transformed into tobacco and 50 days old rice leaves using *Agrobacterium* infiltration^26^,^40^. These leaves were used for pull down assays and rice resistance assay. The total protein was isolated from leaves by crushing them in extraction buffer (composition described in^22^. After centrifugation at cold temperature the protein of interest was isolated from total protein extract by immunoprecipitation using protein A sepharose bound antibody against the tag.

### Total RNA extraction and gene expression analysis

RNA extraction and Real time expression analysis was performed^41^. 2^−ΔΔC^_T_ values were calculated by method described in ref. 42.

For Semi quantitative RT PCR (Semi-Q-RT PCR), reactions were prepared containing cDNA, 10 μM forward and reverse primer, Taq DNA polymerase, buffer and dNTPs. PCR was carried out with 27 cycles and the PCR product was separated on 1.5% agarose gel.

### MAPK activation assay

Total plant protein was isolated from rice leaves overexpressing OsMPK7 and infected with *X. oryzae* by method described above. Concentration of protein extracts were determined using Bradford method. OsMPK7-GFP protein was immunoprecipitated using anti-GFP antibody. Equal amount of beads were loaded onto the 10% SDS polyacrylamide gel and electrophoresis was performed. Proteins were blotted onto the Hybond –C polyvinylidene fluoride (PVDF) membrane (Amersham biosciences, UK). For immunoblotting, the blot was blocked in blocking buffer for overnight, followed with incubation with p42p44 (pTEpY) primary antibody (Cell signaling, USA) for two hours. After washing trice, incubation with HRP conjugated 2° antibody was performed. Signal was detected by treating membrane with Chemiluminescent HRP substrate (Millipore, USA).

### *In vitro* kinase assay

Kinase assay was performed as reported in refs 41 and 43.

### Co-immunoprecipitation assay

Proteins were isolated from *N. benthamiana* leaves transiently expressing OsMPK7-myc and OsMKK3-HA alone and both together. Co-IP was followed from ref. 22.

### Bimolecular fluorescence complementation assay

The cloned constructs in pSPYNE(R)173 and pSPYCE(M) were transformed into onion epidermal cells and *N. benthamiana* by particle bombardment and Agrobacterium infiltration respectively. After 72 hours of incubation, the samples were observed under confocal microscope (Leica TCS SP2, Germany) at wavelength 512 nm excitation and 527 nm emission^40^.

### Rice resistance assay

50 days old rice leaves were infiltrated with Agrobacterium carrying cloned constructs in infiltration medium (10 mM MES hydrate, 10 mM MgCl_2_, 200 μM acetosyringone). After 24 hours, *X. oryzae* in PBS buffer (O.D. 0.7–0.8) was injected into the rice leaves mid-rib 1 cm above Agroinfiltration. After 10 days leaves were observed for the appearance of visible disease lesions.

### Cell death assay

Surface sterilized rice seeds were germinated on Murashige and Skoogs medium and were incubated with *Agrobacterium* suspension carrying cloned constructs for 2–3 hrs at 28 °C followed by transfer on co-cultivation medium for 3 days. Co-cultivated roots were then separated from seedlings, washed with 1X PBS and incubated with *X. oryzae* suspension (O.D. 0.9–1.0) for 5–6 hours. The roots were washed, stained with propidium iodide for 5 min and observed under confocal microscope with absorption/emission of 535/617 nm.

### Bacterial count assay

Overexpressed rice leaves were infected with *X. oryzae* after 24 hrs of Agroinfiltration. After 48 hrs 1 cm segments were cut along the leaves and crushed in 1XPBS solution. Serial dilutions of this extract were made and plated on Peptone sucrose (PS) rifampicin medium.

## Additional Information

**How to cite this article**: Jalmi, S. K. and Sinha, A. K. Functional Involvement of a Mitogen Activated Protein Kinase Module, OsMKK3-OsMPK7-OsWRK30 in Mediating Resistance against *Xanthomonas oryzae* in Rice. *Sci. Rep*. **6**, 37974; doi: 10.1038/srep37974 (2016).

**Publisher's note:** Springer Nature remains neutral with regard to jurisdictional claims in published maps and institutional affiliations.

## Supplementary Material

Supplementary Information

## Figures and Tables

**Table 1:** Function of OsMKK3-OsMPK7-OsWRKY30 module in reducing disease lesions formed after *X. oryzae* infection in rice leaves.

	Treatment^a^	Experiment 1		Experiment 2		Experiment 3	
		No. of leaves^b^	Lesion (cm)^c^	No. of leaves^b^	Lesion (cm)^c^	No. of leaves^b^	Lesion (cm)^c^
MPK7	Control WT+ *X. oryzae*	4/4	13.5	5/5	19.1	4/4	15.1
	Vector+ *X. oryzae*	4/4	10.0	5/5	15.1	4/4	12.1
	MPK7-OE+ *X. oryzae*	4/4	1.3	4/5	1.8	¾	1.0
	MPK7-RNAi+ *X. Oryzae*	4/4	11.2	5/5	17.1	4/4	14.9
MKK3-MPK7	Vector+ *X. oryzae*	5/5	15.4	5/5	18.8	4/4	15.0
	MKK3-OE+ *X. oryzae*	4/5	0.7	3/5	1.1	4/4	1.3
	Vector+ *X. oryzae*	5/5	15.5	5/5	20.1	4/4	16.0
	MPK7-OE+ MKK3-OE+ *X. oryzae*	5/5	0.5	4/5	0.7	¾	0.4
	MPK7-RNAi+ MKK3-OE+ *X. oryzae*	5/5	8.5	5/5	12.8	4/4	8.2
MPK7-WRKY30	Vector+ *X. oryzae*	5/5	17.1	5/5	18.4	4/4	15.1
	WRKY30-OE+ *X. oryzae*	4/5	0.4	4/5	0.5	4/4	0.5
	Vector+ *X. oryzae*	5/5	16.6	5/5	19.7	4/4	14.3
	MPK7-OE+ WRKY30-OE+ *X. oryzae*	5/5	0.2	3/5	0.2	¾	0.3
	MPK7-RNAi+ WRKY30-OE + *X. oryzae*	5/5	8.9	5/5	11.1	4/4	7.5

^a^The mid-rib of 50 days old rice leaves were injected with *Agrobacterium* carrying constructs mentioned in the table above. After 24 hours these leaves were inoculated with *X. oryzae* 1 cm above the point of *Agrobacterium* injection.

^b^Number of leaves showing disease lesion, which were observed after 10 days of *X. oryzae* inoculation.

^c^Average lesion length obtained in leaves infiltrated with empty control vectors and leaves with MPK7-OE, MPK7-RNAi, MKK3-OE, MPK7-OE + MKK3-OE, MPK7-RNAi + MKK3-OE, WRKY30-OE, MPK7-OE + WRKY30-OE and MPK7-RNAi + WRKY30-OE prior to *X. oryzae* inoculation.
