# Supplementary material for: Functional Involvement of a Mitogen Activated Protein Kinase Module, OsMKK3-OsMPK7-OsWRK30 in Mediating Resistance against Xanthomonas oryzae in Rice
Source: Sci Rep. 2016 Nov 29;6:37974. doi: 10.1038/srep37974 (PMC5126639; doi:10.1038/srep37974)
Supplement: Supplementary Information [file srep37974-s1.doc]

**Functional Involvement of a Mitogen Activated Protein Kinase Module, OsMKK3-OsMPK7-OsWRKY30 in Mediating Resistance against *Xanthomonas oryzae* in Rice**

**Siddhi Kashinath Jalmi and Alok Krishna Sinha***

National Institute of Plant Genome Research, Aruna Asaf Ali Marg, New Delhi – 110067, India

*Corresponding Author:

Dr. Alok Krishna Sinha

Staff Scientist VI

National Institute of Plant Genome Research, Aruna Asaf Ali Marg, New Delhi 110067, India

Email: [alok@nipgr.ac.in](mailto:alok@nipgr.ac.in)

Telephone: +911126735188

Fax:+911126741658

**Supplementary Figure Legends**

**Fig. S1.** Analysis of OsMPK7 transcript level in *OsMPK7* overexpressed and silenced rice leaves and roots.

Quantification of OsMPK7 transcript level in (**a**) *OsMPK7* overexpressed and (**b**) *OsMPK7* silenced rice leaves by Q-RT-PCR. Empty vector pCAMBIA1302 transformed leaves were used as control. Actin and ubiquitin were used as internal controls. Six to nine transformed rice leaves (50 days old) were screened in this experiment. Similar result was obtained in three independent experiments.

Semi Q-RT PCR showing expression of *OsMPK7* in (**c**) *OsMPK7* overexpressed rice leaves and (**d**) *OsMPK7* silenced rice leaves. Actin and ubiquitin were used as internal controls. Similar expression was obtained in three independent experiments.

(**e**) Graph representing the percentage of transformed leaves with overexpressed and silenced OsMPK7 expression.

Quantification of OsMPK7 transcript level in OsMPK7 overexpressed and silenced rice roots by (**f**) Q-RT-PCR and (**g**) semi Q-RT PCR. Empty vector pCAMBIA1302 transformed roots were used as control. Similar result was obtained in three independent experiments.

**Fig. S2.** Analysis of OsMPK7 and OsMKK3 transcript level in overexpressed and silenced rice leaves and roots.

Q-RT-PCR to quantify OsMKK3 and OsMPK7 transcript level in transformed rice leaves (**a**) overexpressing *OsMKK3* alone, (**b**) overexpressing *OsMKK3* together with *OsMPK7* and (**c**) overexpressing *OsMKK3* and silenced *OsMPK7*. Empty vectors pSPYCE(M) and pSPYCE(M)+pSPYNE(R)173 transformed leaves were used as control. Actin and ubiquitin were used as internal controls. Six-eight transiently transformed rice leaves (50 days old) were screened in one experiment. Similar result was obtained in three independent experiments.

Semi Q-RT PCR for expression analysis of *OsMPK7* and *OsMKK3* in 50D old transformed rice leaves (**d**) overexpressing *OsMKK3* alone, (**e**) overexpressing *OsMKK3* together with *OsMPK7* and (**f**) overexpressing *OsMKK3* and silenced *OsMPK7*. Empty vectors pSPYCE(M) and pSPYCE(M)+pSPYNE(R)173 transformed leaves were used as control. Similar expression was obtained in three independent experiments. Actin and UBQ5 was used as internal controls.

(**g**) Graph representing the percentage of transformed leaves with overexpressed OsMKK3 and either overexpressed or silenced OsMPK7.

Quantification of OsMKK3 and OsMPK7 transcript level by Q-RT-PCR in transformed rice roots (**h**) overexpressing *OsMKK3* alone and (**i**) overexpressing *OsMKK3* together with either overexpressed or silenced *OsMPK7*. Empty vector pSPYCE(M) and pSPYCE(M)+pSPYNE(R)173 transformed roots were used as control. Similar result was obtained in three independent experiments.

(**j**) Quantification of OsMKK3 and OsMPK7 transcript level by Q-RT-PCR in transformed rice roots overexpressing *OsMKK3* alone and together with either overexpressed or silenced *OsMPK7*. Empty vector pSPYCE(M) and pSPYCE(M)+pSPYNE(R)173 transformed roots were used as control. Actin and UBQ5 was used as internal controls.

(**k**) Immunoblotting of OsMPK7-Myc and OsMKK3-HA confirms the presence of both recombinant fusion proteins in transformed rice leaves (L) and roots (R) overexpressing *OsMKK3* and *OsMPK7*.

**Fig. S3.** Analysis of OsMPK7 and OsWRKY30 transcript level in overexpressed and silenced rice leaves and roots.

Q-RT-PCR to quantify OsWRKY30 and OsMPK7 transcript level in transformed rice leaves (**a**) overexpressing *OsWRKY30* alone, (**b**) overexpressing *OsWRKY30* together with *OsMPK7* and (**c**) overexpressing *OsWRKY30* and silenced *OsMPK7*. Empty vectors pSPYCE(M) and pSPYCE(M)+pSPYNE(R)173 transformed leaves were used as control. Actin and ubiquitin were used as internal controls. Five-seven transiently transformed rice leaves (50 days old) were screened in one experiment. Similar result was obtained in three independent experiments.

Semi Q-RT PCR for expression analysis of *OsMPK7* and *OsWRKY30* in 50D old transformed rice leaves (**d**) overexpressing *OsWRKY30* alone, (**e**) overexpressing *OsWRKY30* together with *OsMPK7* and (**f**) overexpressing *OsWRKY30* and silenced *OsMPK7*. Empty vectors pSPYCE(M) and pSPYCE(M)+pSPYNE(R)173 transformed leaves were used as control. Similar expression was obtained in three independent experiments. Actin and UBQ5 was used as internal controls.

(**g**) Graph representing the percentage of transformed leaves with overexpressed OsWRKY30 and either overexpressed or silenced OsMPK7.

Quantification of OsWRKY30 and OsMPK7 transcript level by Q-RT-PCR in transformed rice roots (**h**) overexpressing *OsWRKY30* alone and (**i**) overexpressing *OsWRKY30* together with either overexpressed *OsMPK7* or (**j**) silenced *OsMPK7*. Empty vector pSPYCE(M) and pSPYCE(M)+pSPYNE(R)173 transformed roots were used as control. Similar result was obtained in three independent experiments.

(**k**) Quantification of OsWRKY30 and OsMPK7 transcript level by Q-RT-PCR in transformed rice roots overexpressing *OsWRKY30* alone and together with either overexpressed or silenced *OsMPK7*. Empty vector pSPYCE(M) and pSPYCE(M)+pSPYNE(R)173 transformed roots were used as control. Actin and UBQ5 was used as internal controls.

**Supplementary Table Legends**

**Table S1.** List of primers used in the study.

**Table S2.** List of probable OsMPK7 protein interacting partners obtained through *in silico* study using STRING 9.0 database.

**Fig. S1**

**
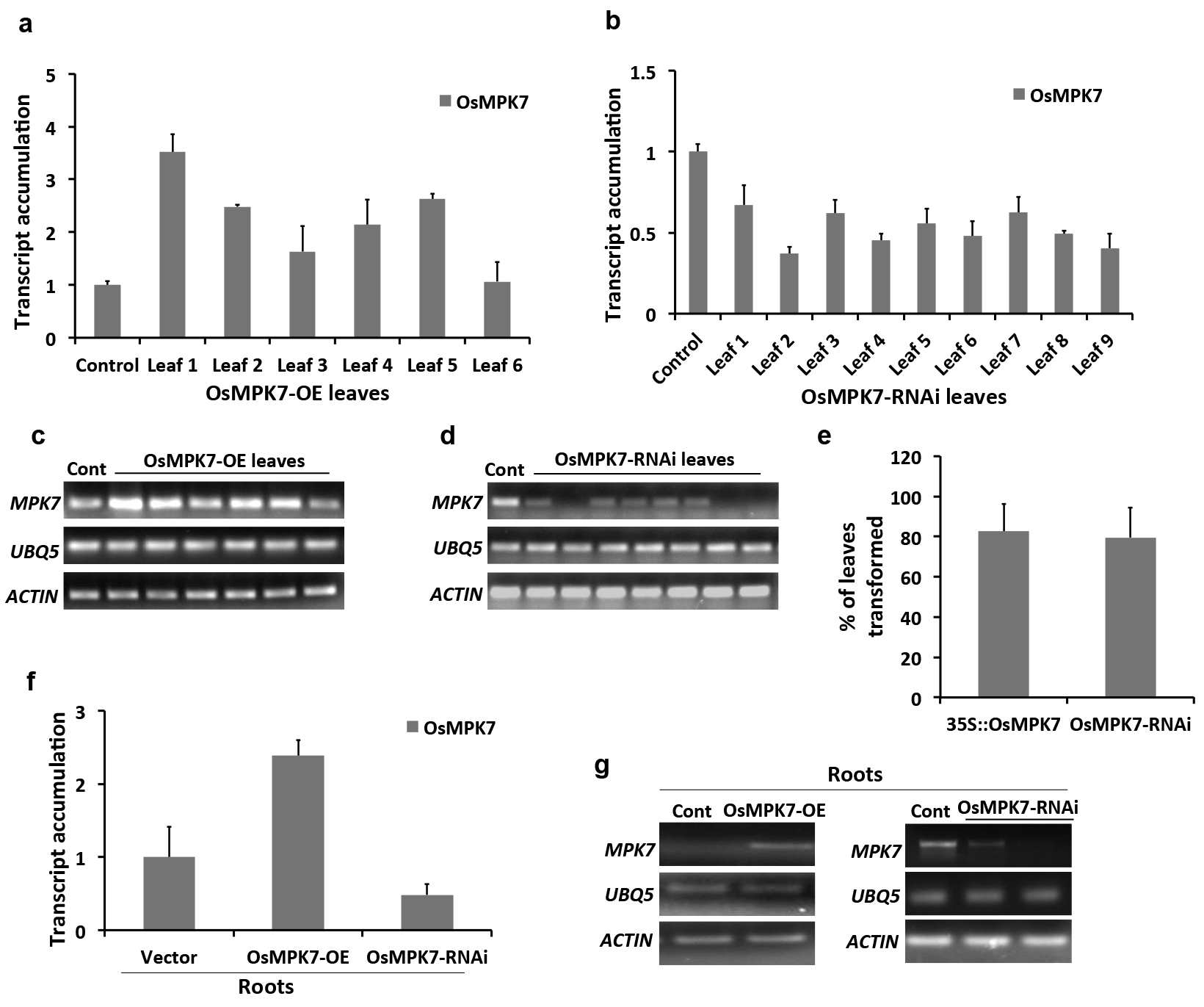
**

**Fig. S2**

**
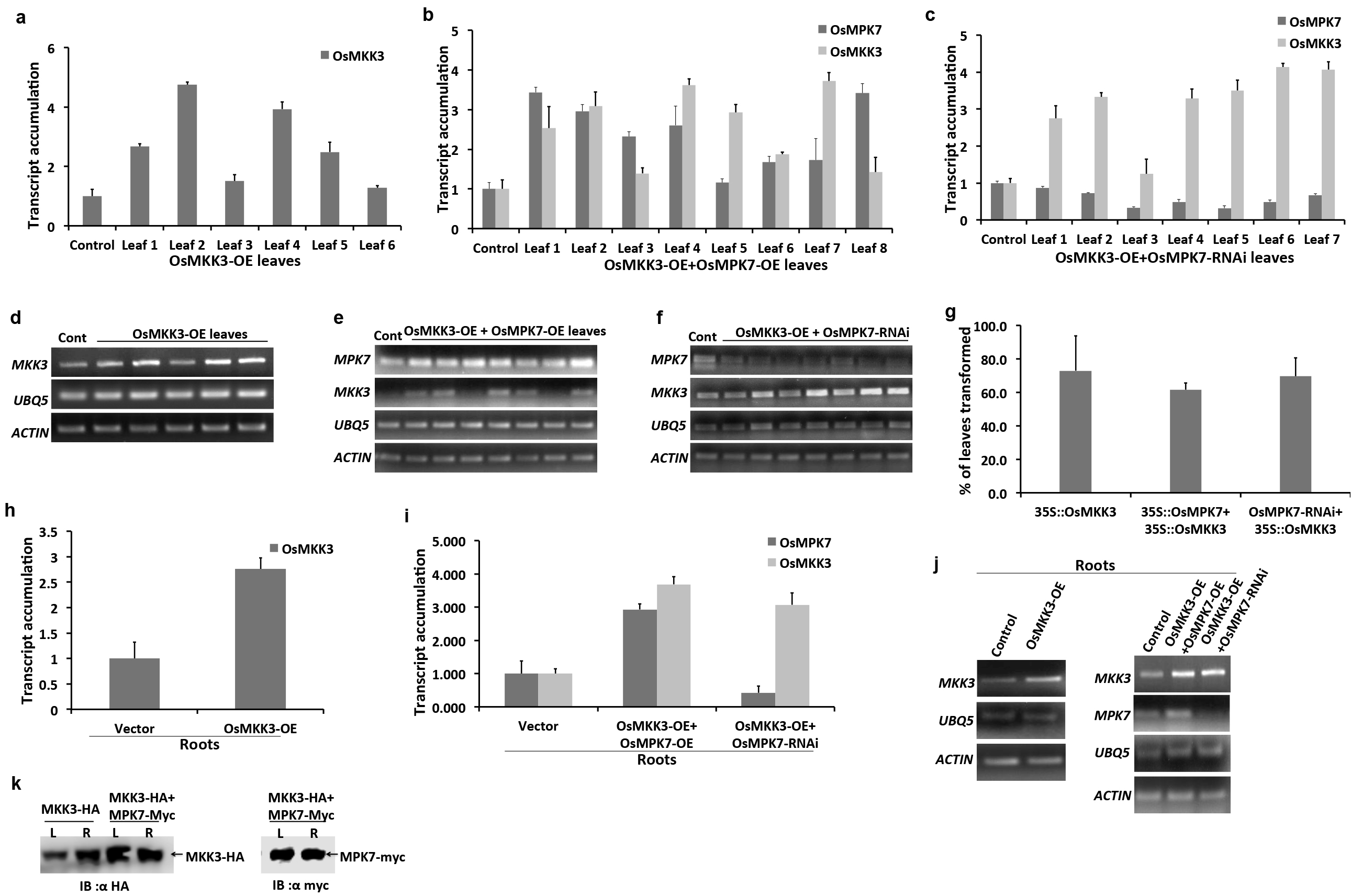
**

**Fig. S3**

**
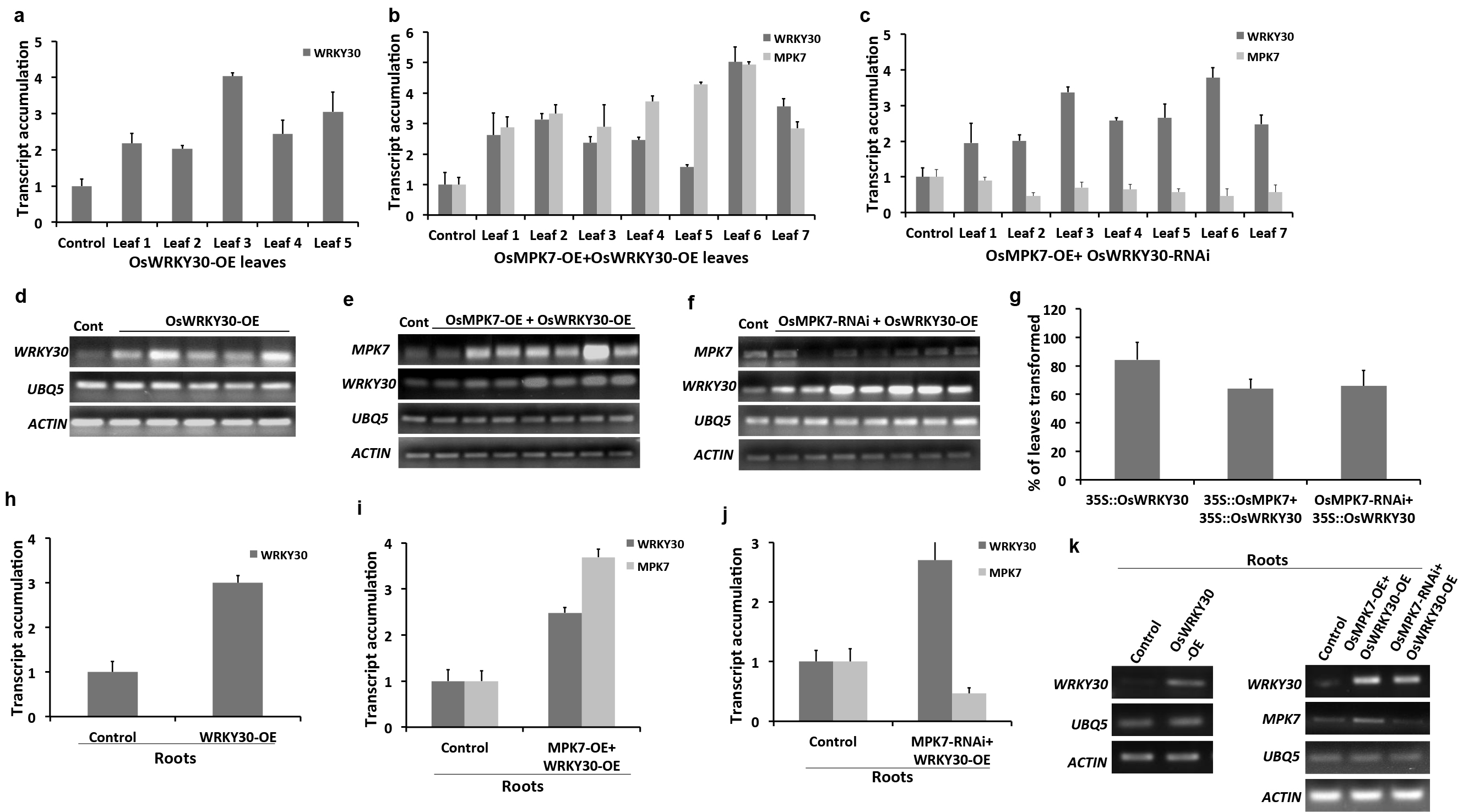
**

**Table S1.** List of primers used in the study.

| **Sr. No.** | **Primer name** | **5’-3’ Sequence** | **Purpose** |
| --- | --- | --- | --- |
| 1. | OsMPK7-pGEX4T-2 Forward | CGGGATCCATGGTGATGATGGTGGACCCTC | Cloning in pGEX4T-2 expression vector |
| 2. | OsMPK7-pGEX4T-2 Reverse | TCCCCCGGGGGATCACATATTCACTCCTGCAACA |
| 3. | OsMPK7-pSPYNE(R)173 Forward | CGCGGATCCATGGTGATGATGGTGGA | Cloning in pSPYNE(R)173 binary vector |
| 4. | OsMPK7- pSPYNE(R)173 Reverse | CGGGGTACCTCACATATTCACTCCTGCAAC |
| 5. | OsMKK3- pSPYCE(M) Forward | CGCGGATCCATGGCGGGGCTCGAG | Cloning in pSPYCE(M) binary vector |
| 6. | OsMKK3- pSPYCE(M) Reverse | CGGGGTACCGGCTTGGATGATGTATAGATCT |
| 7. | OsWRKY30- pSPYCE(M) Forward | CGCGGATCCATGGACGGGACCAACAACCATGG | Cloning in pSPYCE(M) binary vector and pGEX4T-2 expression vector |
| 8. | OsWRKY30- pSPYCE(M) Reverse | CCCCCGGGCATCTGAGGATGCTGCTTTGGCAACC |
| 9. | OsMPK7 real time (RT) Forward | GCTCGCACAAACAACAC | Real time PCR analysis |
| 10. | OsMPK7 RT Reverse | GCCAAGAAGCTCAGCAA |
| 11. | OsMKK1 RT Forward | ACCATCGGCAAATTCCTGAC |
| 12. | OsMKK1 RT Reverse | GAACCAACTGCACGATTCCA |
| 13. | OsMKK3 RT Forward | GTTGAATTCCAGGGTGCATT |
| 14. | OsMKK3 RT Reverse | TTCATGCAAGTAGCGCAAAC |
| 15. | OsMKK4 RT Forward | GGACCATCGCCTACATGAGC |
| 16. | OsMKK4 RT Reverse | GGCGAGTCGGAGTAGCAAAT |
| 17. | OsMKK6 RT Forward | TCCGAGGAAACTGCAGATGA |
| 18. | OsMKK6 RT Reverse | TTTGCGAACTGCCTCTTGAA |
| 19. | OsMPK3 RT Forward | GCTCCAACCAAGAACTGTC |
| 20. | OsMPK3 RT Reverse | AGTCGCAGATCTTGAGG |
| 21. | OsMPK4 RT Forward | CGAGGTCTCCTCCAAGTACG |
| 22. | OsMPK4 RT Reverse | GCGAAGCAGCTTGATTTCTC |
| 23. | OsMPK6 RT Forward | AGGTCACCGCCAAGTACAAG |
| 24. | OsMPK6 RT Reverse | AGCAGCTTGATCTCCCTGAG |
| 25. | OsMPK14 RT Forward | TCCTGAGTTGCTCCTTTGCT |
| 26. | OsMPK14 RT Reverse | CGAGCTTTTGGGTTGTCAAT |
| 27. | PR1b RT Forward | AGAACTACGCCAGCCAGAGAAG |
| 28. | PR1b RT Reverse | AGAAGAGGTTCTCGCCAAGGTT |
| 29. | PR2 RT Forward | CCGGGAGCATCGAGACCTA |
| 30. | PR2 RT Reverse | CTCGTCGCCTCCCTTCTG |
| 31. | PR3 RT Forward | AGGACCCGACAATCTCTTTCAA |
| 32. | PR3 RT Reverse | CCTGGTGCACGTTGTTCATC |
| 33. | PR10 RT Forward | GAGTGGAGGTGAAGGACGAGAT |
| 34. | PR10 RT Reverse | GCCTCGGCGGTCTTGAA |
| 35. | PAL RT Forward | AAGGTGTTCCTCGGCATCAG |
| 36. | PAL RT Reverse | TCCTTGAGGCAGTCGAGCAT |
| 37. | EXLB1 RT Forward | TCCAGCTTTGTGAGACTGTGAATT |
| 38. | EXLB1 RT Reverse | GCCCACACTGCACCATGAG |
| 39. | GDSL lipase like protein RT Forward | CTGTCGAAGCAGGTGGTGTACT |
| 40. | GDSL lipase like protein RT Reverse | CGCCTTCGCCACCATCT |
| 41. | OsNPR3 RT Forward | GCAGAGGACCAGCAAACCAA |
| 42. | OsNPR3 RT Reverse | GCCTTCCGTACATCCTCTCTGA |
| 43. | PI 2-4 RT Forward | GCCATTGAGGCATTGAAGACA |
| 44. | PI 2-4 RT Reverse | CTTTCCAGCATCCCCTTCAAC |
| 45. | RERJI RT Forward | GGATAGTGTCCAAATGAAGCAGATG |
| 46. | RERJI RT Reverse | TGCCGGTCGCCACAAG |
| 47. | ThiC RT Forward | CACATCACCTCCGCCATTG |
| 48. | ThiC RT Reverse | CAGAGAAGTGCAGTGCCAAGAG |
| 49. | TIP RT Forward | GGGATAGGGCCCATGCA |
| 50. | TIP RT Reverse | GAAGAGGAGGGAGAAGGTTAGGA |

**Table S2.** List of probable OsMPK7 protein interacting partners obtained through *in silico* study using STRING 9.0 database.

| **Sr. no.** | **Locus ID** | **Name** |
| --- | --- | --- |
| 1 | LOC_Os05g03865 | LIP19 (bZIP transcription factor) |
| 2 | LOC_Os01g07910 | NADH-Cytochrome b5 reductase |
| 3 | LOC_Os12g07590 | Protein-tyrosine phosphatase domain containing protein |
| 4 | LOC_Os01g07880 | Transcription factor HY5 |
| 5 | LOC_Os01g64000 | OREB1 (ABA responsive factors) |
| 6 | LOC_Os08g38990 | WRKY30 |
| 7 | LOC_Os08g17400 | WRKY89 |
| 8 | LOC_Os11g02520 | WRKY104 |
| 9 | LOC_Os02g36974 | 14-3-3 |
